# MicroRNAs as Biomarkers of Systemic Changes in Response to Endurance Exercise—A Comprehensive Review

**DOI:** 10.3390/diagnostics10100813

**Published:** 2020-10-13

**Authors:** Aleksandra Soplinska, Lukasz Zareba, Zofia Wicik, Ceren Eyileten, Daniel Jakubik, Jolanta M. Siller-Matula, Salvatore De Rosa, Lukasz A. Malek, Marek Postula

**Affiliations:** 1 Center for Preclinical Research and Technology CEPT, Department of Experimental and Clinical Pharmacology, Medical University of Warsaw, 02-097 Warsaw, Poland; ola@soplinska.pl (A.S.); lukaszzareba01@gmail.com (L.Z.); zofiawicik@gmail.com (Z.W.); ceren.eyileten-postula@wum.edu.pl (C.E.); dr.jakubik@gmail.com (D.J.); jolanta.siller-matula@meduniwien.ac.at (J.M.S.-M.); 2 Centro de Matemática, Computação e Cognição, Universidade Federal do ABC, São Paulo 055080-90, Brazil; 3 Department of Cardiology, Medical University of Vienna, 1090 Vienna, Austria; 4 Division of Cardiology, Department of Medical and Surgical Sciences, “Magna Graecia” University, 88100 Catanzaro, Italy; saderosa@unicz.it; 5 Department of Epidemiology, Cardiovascular Disease Prevention and Health Promotion, National Institute of Cardiology, 04-635 Warsaw, Poland; lmalek@ikard.pl; 6 Longevity Center, 00-761 Warsaw, Poland

**Keywords:** microRNA, endurance sport, adaptive changes, cardiac hypertrophy, cardiac fibrosis

## Abstract

Endurance sports have an unarguably beneficial influence on cardiovascular health and general fitness. Regular physical activity is considered one of the most powerful tools in the prevention of cardiovascular disease. MicroRNAs are small particles that regulate the post-transcription gene expression. Previous studies have shown that miRNAs might be promising biomarkers of the systemic changes in response to exercise, before they can be detected by standard imaging or laboratory methods. In this review, we focused on four important physiological processes involved in adaptive changes to various endurance exercises (namely, cardiac hypertrophy, cardiac myocyte damage, fibrosis, and inflammation). Moreover, we discussed miRNAs’ correlation with cardiopulmonary fitness parameter (VO_2max_). After a detailed literature search, we found that miR-1, miR-133, miR-21, and miR-155 are crucial in adaptive response to exercise.

## 1. Introduction

The old Latin saying “mens sana in corpore sano” indicates that the beneficial influence of exercise has been known for centuries. Cardiovascular diseases (CVDs) are the leading cause of death worldwide. According to the World Health Organization (WHO) data, they are responsible for approximately 31% of deaths annually. It is estimated that nearly 80% of premature CVDs are preventable by modification of lifestyle including regular physical activity [1]. Regular exercise of moderate intensity has a beneficial influence in cardiovascular health. It is considered to be one of the most valuable nonpharmacological strategies to prevent and reduce the risk of coronary artery disease and myocardial infarction by up to 50% [2]. According to the European Society of Cardiology, adults should engage in at least 150 min per week (min/week) of moderate intensity or 75 min/week of vigorous intensity aerobic exercise to reduce atherosclerotic cardiovascular disease risk. Furthermore, it is recommended to gradually increase aerobic exercise to 300 min/week of moderate intensity or 150 min/week of vigorous intensity for additional benefits [3].

Endurance training can be described as long-time activity characterized by high dynamic and low to high power load such as swimming, rowing, cycling, running, or a combination of those [4]. From a physiological point of view, the main intent of endurance training is to edge the threshold of activation for anaerobic metabolism and lactate production. High-intensity training demands a sustained 5- to 6-fold increase in cardiac output for prolonged time. Repetitive effort is further compensated by electrical, structural, and functional cardiac adaptation called the “athlete’s heart” which is characterized by an increase in left ventricular (LV) wall thickness, symmetrical increase in both left and right ventricular and atrial capacity size, and often borderline LV ejection fraction [5].

Despite unarguable beneficial influence, endurance exercise can act as a double-edged sword. Intensive exercise in adolescent and young athletes may be rarely associated with the risk of sudden cardiac death (SCD). The combination of LV hypertrophy, enlargement, and/or low ejection fraction can overlap with inherited cardiomyopathies, which are the most common causes of SCD in young sportsmen [6]. For this reason, the differential diagnosis between athlete’s heart and disease using conventional methods remains challenging and new biomarkers are needed to distinguish physiology from pathology.

Over past years, microRNAs (miRNAs) have emerged as potential biomarkers for adaptive changes in response to exercise. MicroRNAs are short noncoding RNAs that regulate post-transcriptional gene expression by inhibiting protein translation or enhancing degradation of messenger RNA (mRNA). MicroRNAs are involved in the development of normal, functional heart tissue. They control cell growth, cell differentiation, apoptosis, and proliferation and are involved in the pathophysiology of cardiovascular pathologies like hypertrophy, fibrosis, and cardiomyocytes’ damage [7].

MicroRNAs comprise a leading class of small RNAs in most tissues. MiRNA genes are located in various genomic contexts, however, the majority of human miRNAs are encoded by introns (noncoding transcripts). MiRNA biosynthesis starts by production of primary miRNA by RNA polymerase II. Then, miRNAs are further processed by a complex consisting of the RNA binging protein, DGCR8 microprocessor subunit, and the endoribonuclease, Drosha. Primary miRNAs are exported to the cytoplasm where they are further processed by endoribonuclease Dicer and subsequently loaded onto Argonaute family proteins to form an effector complex [8].

On the strength of their biochemical stability and the ease of access, circulating miRNAs have aroused the interest as potential biomarkers of various pathological states as well as potential therapeutic targets. Multiple studies have been conducted to evaluate their prognostic value in CVDs [9,10].

Circulating miRNAs are altered in response to acute and endurance exercise and can be engaged in the adaptations to exercise. Previous studies assessed miRNA plasma levels in marathon runners and showed increased levels of some miRNAs after marathon runs and their association with standard fitness parameters [11]. Other studies demonstrated correlations between miRNAs expression and cardiac injury markers such as troponin plasma levels, n-terminal b-type natriuretic peptide (NT-pro-BNP), or creatine kinase-MB (CK-MB). Therefore, they are a potential biomarker of cardiac adaptation processes to exercise [12]. Circulating miRNAs may improve exercise evaluation as well as facilitating the differential diagnosis between adaptive changes and pathology [13]. Therefore, in this article, we aim to review the current knowledge of miRNAs’ involvement in endurance training.

## 2. Article Search Process

Electronic databases Pubmed and Scopus were searched up to September 2020. Original studies were reviewed to assess their relevance to our focus, namely the clinical usefulness of miRNAs as biomarkers of adaptive changes in response to endurance exercise based on human studies. We also investigated review articles and meta-analysis and their secondary references were examined for possible inclusion. Papers describing strength exercises were excluded from our review.

The following search syntax was used: “Search (“micrornas” [MeSH Terms] OR “mir” [MeSH Terms] OR “mirna” [MeSH Terms] OR “circulating miRNA” [MeSH Terms] OR “circulating microRNA” [MeSH Terms]) AND (“endurance training” [MeSH Terms] AND (“adaptation” [MeSH Terms] OR “change” [All Fields]) Filters: Humans. Our search was limited to human studies and did not exclude studies on the basis of ethnicity.

## 3. Results

### 3.1. MicroRNAs and Adaptive Cardiac Hypertrophy Versus Hypertrophic Cardiomyopathy

**Figure 1:**
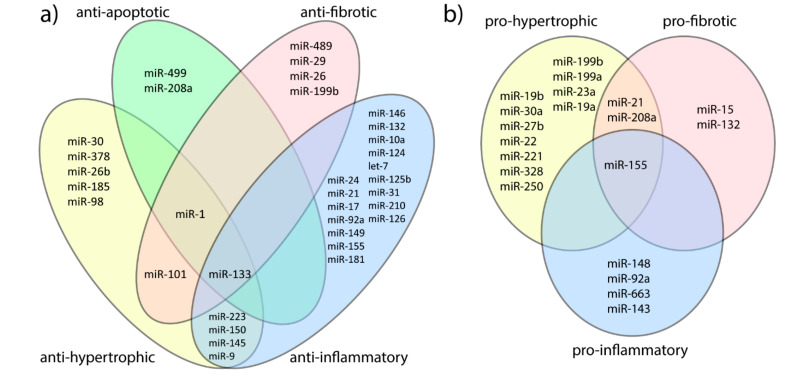
The potential role of microRNAs in cardiac remodeling. (**a**) MicroRNAs associated with ibeneficial response to endurance exercise. (**b**) MicroRNAs associated with pro-inflammatory, pro-fibrotic and pro-hypertrophic processes in cardiac remodeling. miR, microRNA.

Mild myocardial hypertrophy is considered as one of the structural adaptations to endurance training. Well-trained endurance athletes display an increased wall thickness and relevant dilatation of LV obtained mostly by eccentric hypertrophy. This allows increasing the cardiac output and maximal oxygen uptake (VO_2max_) and subsequently improves physical fitness [14]. Numerous studies aimed to correlate miRNAs and hypertrophy and allowed a distinction between anti- and prohypertrophic miRNAs (see Figure 1) [15]. It is important also to distinguish adaptive cardiac hypertrophy from hypertrophic cardiomyopathy (HCM) and find the differences in miRNA expression.

Several studies evaluated the involvement of a number of miRNAs in cardiac hypertrophy, however, the results are conflicting [11,16,17,18]. Moreen et al. investigated a relation of miRNAs with conventional biochemical, cardiovascular, and performance indices in endurance athletes before, immediately after, and 24 h after a marathon run. After the marathon run, significant increases of miR-1, miR-133a, miR-206, miR-208b, and miR-499 were observed. MiR-133a was positively related to the thickness of the intraventricular septum in echocardiography. Interestingly, the expression of miR-1, miR-133a, miR-206 was correlated to themselves (*p* < 0.001), whereas no such correlation was found in the case of miR-208b and miR-499. There is a possibility that the difference occurred due to the stronger skeletal muscle relation of miR-1, miR-133a, and miR-206 than the other measured miRNAs [11]. MiR-1, miR-133, and miR-206 belong to a novel group of miRNAs known as myomiRs. MyomiRNAs are described as striated muscle-specific or muscle-enriched miRNAs that regulate muscle development and homeostasis. They are involved in myoblast proliferation and differentiation as well as muscle regeneration [19]. Interestingly, expression of precursors of the mentioned myomiRs is upregulated in elderly people compared to younger ones. Nevertheless, this difference can no longer be observed while analyzing mature forms of myomiRs [20].

**Figure 2:**
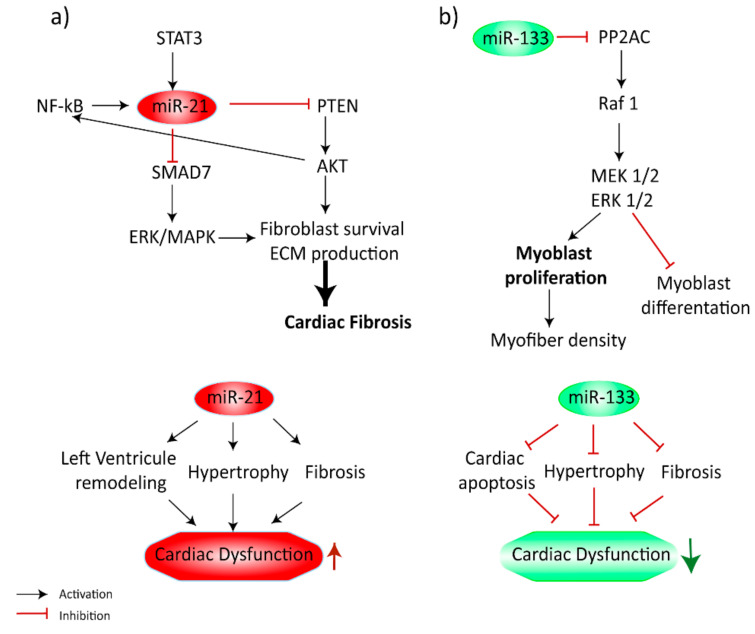
Regulation of cardiac remodeling by microRNAs and the functions of their target mRNAs. MAPK/ERK pathway plays a crucial impact on signal transduction for myosin growth. PI3K/AKT signaling pathway regulates cell proliferation, metabolism, survival, and angiogenesis and controls the development and transformation of left ventricular hypertrophy. The ERK signaling pathway, a member of the MAPK family, comprises a cascade of a series of successively acting kinases. (**a**) The role of miR-21 in cardiac remodeling. Regulation of ERK is mediated by miR-21 via inhibiting PTEN and SMAD7 gene expression. (**b**) The role of miR-133 in cardiac remodeling. MicroRNA-133 plays a role in determining cardiomyocyte hypertrophy by targeting PP2AC. Abbreviations: miR, microRNA; STAT3, signal transducer and activator of transcription 3; NF-κB, nuclear factor-κB; PTEN, phosphatase and tensin homolog; ERK, extracellular signal-regulated kinase; MAPK, mitogen-activated protein kinase; PP2AC, protein phosphatase 2A; Raf-1, Raf-1 proto-oncogene, serine/threonine kinase.

In another study, Ramos et al. hypothesized that miRNAs exhibit dose–response correlation with varying levels of exercise intensity and duration (see details in Table 1). The results showed that miRNAs can be divided into three different expression patterns: (1) responsive, dose-dependent with miR-1 dependent on exercise intensity, and miR-133a, miR-222 dependent on duration of exercise, (2) responsive with no significant dose responsiveness like miR-24, miR-146a, and (3) nonresponsive regardless of intensity or duration of the training (i.e., miR-21, -210). Moreover, using an animal model, it was found that miR-133a correlated positively with increased intraventricular septal thickness and high expression of serum response factor transcripts which play key role in myocyte proliferation and cardiac hypertrophy. Despite the antihypertrophic effect of miR-1 and miR-133a, their plasma level corresponds to the active heart remodeling by a decrease of its intracellular concentration, which may weaken the inhibition effect, activate gene programming, and start the adaptation process [17]. Mooren et al. showed both miR-1 and -133 to be upregulated, which may suggest a similar expression pathway as it was described previously [11,21]. However, in Ramos et al., the gap in expression of those miRNAs was observed. The miR-1 elevation was dependent on the higher intensity of training and was much more increased than miR-133, whereas miR-133 was significantly related to the longer duration of training, which had weaker effect on miR-1 expression [17]. Contradistinctively, Fernandez et al. showed miR-1 correlation with longer exercise (i.e., marathon run) [22]. The difference between studies may occur due to the nature of endurance exertion during marathons, which presents high intensity and long duration features, therefore, simultaneous elevation of miR-1 and miR-133. Additionally, signaling pathways linked to the miR-1 and miR-133 upregulation such as MAPK ERK1/2 or myogenic factors like MyoD or myogenin could be activated by different muscle exertion features, which may result in divergent miRNAs plasma level elevation (see Figure 2) [23,24]. It was shown that ERK1/2 is activated by a hypoxic environment and increases miR-133 expression in cardiomyocytes [23]. Importantly, it was shown that myogenic factors such as myoD and myogenin may regulate the expression of muscle-specific miRNAs, including miR-1, miR-133a/b, and miR-206 in biogenesis and these miRNAs were upregulated during myogenesis [25,26]. On the other hand, Denham et al. [18] demonstrated contrary results; downregulation of miR-1 and miR-133 expressions after acute treadmill exercise was observed. In addition, Baggish et al. [16] detected no miR-133 elevation at the baseline and after the acute exercise at the start and at the end of the 90-day sustained training program. This observation may correspond to the previous finding, as the acute exercise protocol was performed on an upright cycle ergometer and was relatively short. Different results could be obtained due to different features of participants and the training program. However, there is also one relevant difference: the participants have undergone the acute exertion on treadmills not by running. The reason for contrary results can be due to different types of acute exercise. More studies should be conducted to determine the difference in miRNA expression after varied types of endurance exertion.

It is worth mentioning that miR-1 and miR-133 were previously described to be significantly downregulated in heart tissue in patients with HCM [27]. Similar to the endurance athletes, HCM patients showed a significantly increased level of miR-133, but surprisingly, no significant result was observed for miR-1 [28]. This finding may suggest that the poverty of in-tissue miR-1 and miR-133 is important for development of both adaptive hypertrophy and HCM. However, the difference of miR-1 concentration suggests that in the pathological state, miR-1 expression is downregulated in cardiomyocytes, whereas in adaptive hypertrophy, it is probably stable or induced but there is a loss due to sarcolemma damage. This hypothesis should be investigated in the future in athletes and also elderly people, who were proven to develop muscle hypertrophy in response to the training [29].

Importantly, out of 17 articles which aimed to analyze the importance of miRNAs in endurance training, only one study used mRNA–miRNA targeting in silico prediction tool by using bioinformatic analysis via KEGG (Kyoto Encyclopedia of Genes and Genomes) to identify possible molecular pathways. Interestingly, based on functional enrichment analysis, the study identified 31 molecular pathways (enriched with the targets of the miRNA profiles) after a 10 km run, and 61 molecular pathways after the marathon races. These molecular pathways were relatively associated to heart physiology and pathophysiology, including cardiac hypertrophy, cardiac remodeling and function, fibrosis, response to injury, and cell survival and proliferation, which suggests that endurance training may be linked to not only physiological adaptive cardiac hypertrophy, but also pathophysiological cardiac hypertrophy [30].

**Table 1:** Characteristics of microRNA studied in endurance sports.

First Author	miRNAs	Material	Exercise	Training Protocol and Samples Collection	Methodology	Subjects	Results (*p* < 0.05)
Alack 2019 [31]	miR-24, miR-27a, miR-21, miR-15a, miR-23a, miR-221, miR-125b	Leukocytes	-	At rest	qRT-PCR, miRNAeasy Mini kit, TaqTM Universal SYBR Green Supermix	13 trained triathletes and marathon runners (VO_2max_ > 59 mL/kg × min) and 12 untrained healthy controls (VO_2max_ < 45 mL/kg × min)	Endurance athletes: downregulated: miR-21, miR-23a
Backes 2014 [32]	1205 different miRNAs	Whole blood	Cycling	Before and after exhaustive exercise on cyclic ergometer in each group	Microarray, qRT-PCR miScript SYBR Green	12 elite endurance athletes (6 males, 6 females; 10 triathletes, 2 cyclists) and 12 age- and sex-matched controls; included 8 athletes and 8 controls	Endurance/control after exercise miR-181a, miR-320b were decreased in athletes
Baggish 2011 [16]	miR-20a, miR-210, miR-221, miR-222, miR-328, miR-21, miR-146a, miR-21, miR-133a, miR-21, miR-146a, and miR-210	Blood (plasma)	Cycling	At rest and during acute exhaustive exercise testing on upright cycle ergometer, before and after a 90-day period of aerobic exercise training	qRT-PCR	10 competitive male rowers (*n* = 10, age = 19.1 ± 0.6 years)	Elevated by acute exercise before and after sustained training: miR-146a, miR-222 elevated by acute exercise before but not after sustained training: miR-21, miR-221 elevated after sustained training: miR-20a nonresponsive: miR-133a, miR-210, miR-328
Baggish 2014 [12]	miR-1, miR-133a, miR-499-5p, miR-208a, miR-126, miR-146a	Blood (plasma)	Running	At rest, immediately after marathon and 24 h after	qRT-PCR, TaqMan miRNA	21 healthy male marathon runners	Upregulated after the race: miR-126, miR-1, miR-133a, miR-499-5p, miR-208a, miR-146a
Bye 2013 [33]	miR-210, miR-21, miR-125a, miR-652, miR-151, miR-29a, Let-7d, miR-222	Blood (plasma)	VO_2max_ test	Before the start of the exercise test	qRT-PCR	Screening cohort: 12- high VO_2max_, 12- low VO_2max_ validation cohort: 38- high VO_2max_, 38- low VO_2max_	Low VO_2max_ group: upregulated: miR-210, miR-222, miR-21 (with only males)
Danese 2018 [34]	miR-133a, miR-206	Blood (plasma)	Half-marathon	Before and immediately after the half-marathon—21.1 km	qRT-PCR, TaqMan MicroRNA assay	28 middle-aged, recreation athletes (11 women and 17 men; mean age, 46 years)	Elevated after the half-marathon run: miR-133a and miR-206
Fernandez-Sanjurjo 2020 [22]	Global miRNA screening (752 miRs)	Blood (plasma)	Running	Before and immediately after: 10 km race, half-marathon, and marathon	qRT-PCR	9 runners	After 10 km run Upregulated: miR-199b-5p, miR-424-3p, miR-33a-5p, miR-551a, miR-1537, miR-223-5p, miR-1260q, let-7b-3p, miR-150-5p, miR-423-5p, miR-223-3p, miR-345-5p, miR-505-3p Downregulated: miR-346 After half-marathon: Upregulated: miR-425-3p, miR-33a-5p, miR-338-3p, miR-339-5p, miR-106b-3p, miR-502-3p, miR-27a-3p, miR-660-5p, miR-505-3p, miR-100-5p, miR-22-3p, miR-30e-5p, miR-497-5p After marathon: Upregulated:miR-1972, miR- 940, miR-424-3p, miR-130b-5p, miR-223-5p, miR-145-3p, miR-181c-30, miR-501-3p, miR1260a, miR675-3p, miR345-5p, miR-424-5p, miR-1-3p, miR-34a-5p, miR-629-5p, miR-30a-5p, miR-148a-3p, miR-596, miR-10b-5p, miR-30d-5p, miR-320d Downregulated: miR-192-5p, miR-20b-5p, miR-103a-3p, miR-106b-5p, miR144-3p, miR-665, miR-486-3p
Gomes 2014 [13]	miR-1, miR-133a, miR-206	Blood (plasma)	Half-marathon	Before warm-up and up to 10 min after the run	qRT-PCRTaq Man miRNA	5 male recreational runners	Upregulated: miR-1, miR-133a, miR-206
Gonzalo-Calvo 2018 [30]	Panel of 74 c-miRNAs	Blood (plasma)	10 km, half-marathon, marathon	Before and after (0 h, 24 h, 72 h): 10-km, half-marathon, and marathon separated by one-month break	qRT-PCR miScript SYBR Green	9 healthy, highly trained middle-aged amateur subjects	10 km run: immediately after – increased miRNAs: miR-132-3p, miR-150-5p, decreased miRNAs: miR-103a-3p and miR-139-5p 24 h after – decreased miRNA: miR-590-5p Marathon run: immediately after – increased miRNAs: miR-21-5p, miR-27a-3p, miR-29a-3p, miR-30a-5p, miR-34a-5p, miR-126-3p, miR-142-5p, miR-143-3p, miR-195-5p, miR-199a-3p 24 h after– decreased miRNAs: miR-25-3p, miR-29b-3p, miR-30b-5p, miR-106b-5p, miR-107, miR-497-5p downregulated immediately after and remained downregulated for 24 h: miR-103a-3p and miR-375-5p
Denham 2016 [18]	miR-1, miR-133a, miR-181a, miR-486, and miR-494	Whole blood	Running-sprint	Before and after 4 weeks (thrice weekly) of sprint interval training and a single bout of maximal aerobic treadmill exercise	qRT-PCR, TaqMan miRNA	67 endurance athletes and 61 healthy controls; 19 young men—acute exercise trial	Endurance athletes, increased: miR-1, miR-486, and miR-494 after endurance training Healthy, young men decreased: miR-1, miR-133a, and miR-486 immediately after maximal aerobic exercise
Kern 2020 [35]	Global miRNA	Blood (plasma)	Running	Before, after 8 weeks of endurance training, after 8 weeks of wash-out phase, and after another 8 weeks of endurance training	Microarray	23 healthy untrained volunteers	Most important miRNA associated with VO_2max_ Cluster 1:miR-4465, miR-5581-5p, miR-6879-5p, miR-6869-5p Cluster 2: miR-7975 Cluster 6: miR-326-5p, miR-502-5p, miR-502-3p, miR-340-5p
Kravinen 2019 [36]	miR-21, miR-26, miR-126, miR-146, miR-221, miR-222	Blood (serum and extracellular vesicles, EV) and sweat (EV)	Cycling	Sweat collection during, blood collection before and after each protocol: (1) maximal aerobic capacity test (2) anaerobic threshold, and (3) aerobic threshold (AerT) tests. Sauna—control	qRT-PCR, miRNAeasy Mini kit, miScript II RT Kit	8 healthy trained subjects (all protocols)	Elevated In sweat: All endurance exercise: miR-26 Protocol 3 vs. control: miR-21 In serum EVs: Protocol 2 vs. control: miR-21, miR-222
Mooren 2013 [11]	miR-1, miR-133, miR- 206, miR-499, miR-208b, miR- 21, and miR-155	Blood (plasma)	Marathon	2 days before in the morning, directly after, and 24 h after a public marathon run	qRT-PCR TaqMan miRNA	14 male endurance athletes	Increased after race: miR-1, miR-133a, miR-206, miR-208b, miR-499 Elevated 24 h after race: miR-1, miR-133a, miR-206
Nielsen 2013 [37]	global miRNA (742 miRNA)	Blood (plasma)	Cycling	Before (at rest) and immediately after 1 h, post 1 h, post 3 h an acute exercise training (60 min cycle ergometer exercise at 65% of Pmax) and following 12 weeks of endurance training (cycle ergometer with frequency of 5 times per week for 12 weeks)	Microarray, RT-PCR, miScript SYBR green and ROX, Exiqons miRNome panel V.2, ViiA7 Sequence Detection	13 healthy men—acute exercise training, 7 healthy men—endurance training	Immediately after: all downregulated: miR-106a, miR-221, miR-30b, miR-151-5p, Let7i, miR-146a, miR-652, miR-151-3p upregulated 1 h–3 h: after 1 h: miR-330-3p, miR-223, miR-139-5p, miR-143, miR-145, miR-424 after 3 h: miR-1, miR-424, miR-133a, miR-133b after 12-week training: a) upregulated: miR-103, miR-107 b) downregulated: miR-342-3p, Let-7d, miR-766, miR-25, miR-148a, miR-185, miR-21, miR-148b, miR-133a, miR- 92a, miR-29b
Ramos 2017 [17]	miR-21, miR-210, miR-24, miR-146, miR-1, miR-133, miR-222	Blood (plasma)	Running	Two studies: 1) controlled intensity 1-week intervals at 3 intensities (6,7,8 miles/h) and final 5-mile test 2) duration test speed 7 miles/h, 30,60, 90 min duration, final 5-mile treadmill run. Blood samples collected immediately after treadmill running	qRT-PCR, TaqMan miRNA	26 healthy young men—12 in intensity trial and 14 in duration trial	Elevated in both groups and not intensity- or duration-dependent: miR-24, miR-146a Elevated and intensity-responsive: miR-1 Elevated and duration-responsive: miR-133, miR-222
Uhlemann 2014 [38]	miR-126, miR-133	Blood (plasma)		Three studies regarding endurance exercise: Study 1: maximal symptom–limited exercise test, Study 2: bicycling for 4 h, Study 3: running a marathon	qRT-PCR, TaqMan miRNA	Study 1: 13 healthy participants, Study 2: 12 healthy well-trained men, Study 3: 22 male middle-aged marathon runners with no history of coronary artery disease	Study 1: increased miR-126 at maximum power Study 2: increased miR-126 Study 3: increased miR-126 and miR-133
Yin 2020 [39]	miR-1-3p, miR-133a-3p, miR-133b, miR-206	Blood (plasma)	Running	Before, immediately after, and 24 h after 8 km run	qRT-PCR	18 healthy trained young men	Immediately after run elevated: miR-1–3p, miR-133a-3p, and miR-133b 24 h after run: elevated: miR-133a-3p

Abbreviations: microRNA, miR; high-intensity interval training, HIIT; maximal oxygen uptake, VO_2max_; hour, h; maximal power, P_max_; minutes, min; second, s; *p* < 0.005; extracellular vesicles (EV).

### 3.2. MicroRNAs and Cardiomyocytes Damage

It is well known that excessive sustained endurance exercises may lead to increased plasma levels of troponin-I, creatine kinase myocardial band (CK-MB), myoglobin, and B-type natriuretic peptide (BNP), but the cause of this elevation is not clearly understood. Those biomarkers reflect the myocardial cells damage and loss of integrity of their desmosomal connections [40]. In addition, it was shown that elevated cardiac biomarkers such as high-sensitivity cardiac troponin T (hs-cTnT), the N-terminal prohormone of brain natriuretic peptide (NT-proBNP), return to their normal levels within 72 h after excessive exercise such as a marathon without complications [41]. It suggests that excessive endurance exercises can cause temporary, reversible cardiomyocytes sarcolemma damage. Exhaustive endurance exertion can also elevate the plasma levels of muscle-specific miRNA, which are abundantly present in the cardiomyocytes (Figure 1) [42]. In line with that, miR-499, miR-208, miR-133, and miR-1 are reflecting cardiomyocyte damage, and miRNA-1, miR-133, miR-499, and miR-208 possess antiapoptotic function, whereas miR-34 is proapoptotic [43,44,45].

In the study, running induced overexpression of miR-1-3p, miR-133a-3p, miR-133b-3p in young healthy adults. Moreover, miR-133a-3p was still elevated after 24 h of rest, whereas miR-1-3p and miR-133b-3p returned to their baseline. Levels of miR-1-3p and miR-133a-3p were correlated with myoglobin at 24 h after the run [39]. Baggish et al. showed that the concentration of cardiac-tissue-specific circulating miR-208a was significantly upregulated before, but it was substantially decreased 24 h after completing the marathon. Additionally, the conventional biomarkers of the cardiac injury such as hs-cTnT, hs-cTnI, and NT-pro-BNP were increased immediately after the run and remained elevated at the second time point; however, no correlation was found with miR-208a [12]. Similarly, in another study, there was no correlation between cardiac-specific miR-208, as well as miR-1, miR-133a, and miR-499, and the cardiac biomarkers (i.e., CK-MB, troponin T and I, and pro-BNP) measured after the marathon run [11]. Danese et al. found the elevation of miR-133a, miR-206 and CK, hs-cTnT levels after completing a half-marathon run but again no correlation was found. Interestingly, the highest elevation was detected in the miR-133 and hs-cTnT plasma levels, 7.5-fold and 4.2-fold, respectively. The enhanced plasma value of miR-133a may be interpreted as a potential physiological response to high-intensity and/or prolonged exercise, probably aimed to facilitate immediate regeneration or recovery of cardiac and skeletal muscle tissues [34].

All studies showed a significant elevation of specific miRNAs in response to the endurance exercise. MicroRNA-1, miR-133a/b, miR-208, and miR-499 were significantly upregulated immediately after the excessive endurance exertion, however, there was no correlation with the conventional cardiac damage markers at this time point. This lack of correlation may be explained by the different pattern of release into the circulation after cardiomyocyte injury. In line with that, one study found a correlation of miR-1a-3p and miR-133a-3p with myoglobin at 24 h after exercise, which indicates a faster release of those miRNAs than myoglobin, but a similar maintenance in plasma. miRNAs should be considered not only as the direct muscle damage biomarkers but also as indicators of reparative processes in response to external stimulus such as stress. In this scenario, the reason could be the upregulation of miRNA concentration is not correlated with the classic cardiac damage biomarkers [34]. The value of the above-mentioned miRNAs as cardiomyocytes damage or reparative processes biomarkers should be assessed in future studies. In particular, it is of key importance to understand whether a panel of circulating miRNAs might be able to differentiate hypoxic myocardial damage from myocardial injury induced by overload.

### 3.3. MicroRNAs and Fibrosis

Endurance exertion requires a significant increase of cardiac output usually for several hours, thereby enforcing prolonged and high-degree stress to all myocardial structures. This might lead to the positive cardiac training adaptations, but there is also a possibility of pathophysiological cardiac remodeling processes. Nowadays, still there is a discussion in the literature whether myocardial fibrosis may occur in elite endurance athletes due to regular excessive training or such an effect does not exist [46,47]. The fibrosis is a well-known risk factor for the major adverse cardiac events (MACE) and is specifically strongly correlated to the increased risk of arrhythmias [48]. Biomarkers have been established which are correlated with the cardiac fibrosis process, including matrix metalloproteinase (MMP)-1,-2,-3,-8,-9, tissue inhibitor of metalloproteinase (TIMP)-1,-4, TGFbeta1, growth differentiation factor 1 (GDF1), connective tissue growth factor (CTGF), osteopontine, periostine, galactine-3, ST2, and miRNAs [49]. Fibrosis-involved miRNAs, such as miR-1, miR-133, miR-26, miR-29, miR-21, miR-34 can be distinguished into those which are expressed mainly in fibroblasts like miR-29 and miR-21, and the rest of them which are expressed in different types of cells. Moreover, some miRNAs may play an important role as an antifibrotic or profibrotic regulator (Figure 1) [15,50,51].

One of the latest studies analyzed the expression of several miRNAs in response to different doses of endurance running. A significant increase of galectin-3 secretion (a prognostic biomarker in patients with heart failure) was observed immediately after a 10 km run, a half-marathon, and a marathon in a dose–response manner. Also, plasma expression of miR-21-5p was increased twofold, miR-29a-3p fourfold, and miR-34a-5p fourfold after a marathon compared to the baseline, however, all measured parameters returned to baseline values after 24 hours [30]. It may suggest that prolonged myocardial stress during a long endurance exertion could induce signaling towards myocardial fibrosis presented by galectine-3 and miR-21 upregulation. However, antifibrotic miRNAs such as miR-29a, miR-34a were also highly expressed, probably possessing a preventive role. Finally, the study may indicate that an endurance exertion-related fibrosis is rare in amateur athletes due to a preserved balance of pro- and antifibrotic agents. On the contrary, a previously described study may indicate that no profibrotic signaling was present in participants after acute endurance exertion, as fibrosis/inflammation-related miR-21 and miR-155 showed no significant response to exercise [11]. This finding could have occurred due to the features of participants. The study did not determine precisely how well trained runners were, which may indicate that most of them might have been amateur athletes. Furthermore, another study demonstrated that in elite runners, only miR-26 plasma expression was decreased, but no significant difference was observed in nonelite runners [52]. It may indicate that well-trained endurance athletes express weaker antifibrotic reaction after the exertion. It was hypothesized that it may predispose to the occurrence of myocardial fibrosis and arrhythmias like atrial fibrillation in some elite endurance athletes [53]. Another study showed upregulation of miR-29c, miR-222, and TGF-β1 mRNA after a high-intensity interval training program (for details, see Table 1) [54]. As miR-29 is considered to prevent fibrosis by targeting the TGFβ/SMAD3 signaling pathway, TGFβ mRNA expression after the training was evaluated and compared to the miRNA expression [55]. The results may implicate that trained endurance athletes express induced profibrotic signaling through the TGFβ pathway after acute exertion compared to the untrained ones.

Hence, we hypothesize that amateur endurance athletes present no or weak pro-/antifibrotic reaction after acute exertion, while well-trained ones express significant pro-/antifibrotic reaction and finally elite athletes show significant pro- and, in some cases, weaker antifibrotic response, however, due to an insufficient number of participants, such a conclusion should be drawn with caution. All studies showed that miRNAs are emerging important biomarkers of myocardial fibrosis in endurance athletes and should be further investigated. Moreover, regular measurements of c-miRNAs levels during a training program in professional endurance athletes may give crucial information for training customizations and myocardial fibrosis prevention.

### 3.4. MicroRNAs and Inflammatory Response

Inflammation is considered as a key factor of atherosclerosis, the leading CVD disease. It was proven that oxidative stress in vascular endothelial cells leads to accumulation of macrophages and increased production of inflammatory markers like IL-6, IL-1, and TNF-alpha [56]. Depending on the form, exercise seems to have a different influence on the immune system. Endurance training shows a tendency to upregulate the inflammatory biomarkers after an acute bout of exercise and downregulate in long-term programs, however, a previous systematic review on this topic failed to confirm these observations [57].

MicroRNAs are considered as potential biomarkers of inflammation and can be divided into anti-inflammatory and proinflammatory (see Figure 1) and they can correlate with classical markers of inflammatory response (such as leukocyte count, IL-6, IL-10, c-reactive protein-CRP, etc.) [9,58]. Gonzalo-Calvo et al. assessed the classical inflammatory and inflammation-related miRNA response to a 10 km run and a marathon [30]. Total leukocyte and neutrophil counts were elevated after the 10 km run and after the marathon, however, the increase was more pronounced in the marathon group. Furthermore, significant differences were observed in the miRNA expression pattern (after 10 km run, miR-150-5p levels were significantly increased, and after the marathon, let-7d-3p, let-7f-2-3p, miR-29-3p, miR-34a-5p, miR-125b-5p, miR-132-3p, miR-143-3p, miR-148-3, miR-223-5p, miR-424-3p, and miR-424-5p) (for details, see Table 1). Interestingly, miR-150-5p was positively correlated with leukocyte and neutrophil counts after the 10 km run. MiR-150-5p was shown to play an anti-inflammatory role by inhibiting the activation of PI3K and Akt and subsequently the NF-κB pathway detected by in silico analysis, as described by Grabarek et al. [59]. Let-7f-3p was positively correlated with hs-CRP, and miR-29-3p was negatively correlated with IL-10 levels after the marathon. The study suggests that acute endurance exercise induces a dose-dependent circulating inflammatory miRNA response. This might be a reason for the difference in magnitude of the inflammatory response between the race distances. Furthermore, the marathon run seemed to induce higher expression of let-7 family members involved in initiation and development of inflammatory response. Also, Baggish et al. found that inflammation-specific miR-146a, but not hs-CRP, was significantly elevated after the marathon run and returned to baseline value within 24 h (for details, see Table 1). This different temporal pattern of miR-146a rise in comparison to hs-CRP emphasizes a potential role of the miRNAs as novel marker of exercise physiology [12].

In another study using cycle-ergometer expression of two inflammation-related miRNAs in response to acute 60 min long endurance exercise, miR-146 and let-7i were downregulated (for details, see Table 1) [37]. Interestingly, let-7d, miR-21, -29b, -148 expressions were significantly lower after 12-week training. The following observations indicate that both small-dose acute exercise and long-term training may induce an anti-inflammatory response, irrespective of endurance exercise form [30,37]. This is in line with the latest observation where the expression of miR-146 showed the tendency to be increased after different cycling training protocols [36]. Furthermore, a study by Backes et al. showed significantly lower levels of proinflammatory let-7c in endurance athletes compared to controls on baseline [32]. It indicates that regularly training sportsmen have lower immune system reactivity which is in line with de Gonzalo-Calvo’s conclusion that long-term endurance sports induce anti-inflammatory response [46]. A recent study showed that lymphocytes adapt to repetitive endurance exercise by increasing their resistance to apoptosis by upregulation of antiapoptotic and downregulation of proapoptotic miRNAs (especially miR-221) [31]. The alterations in apoptotic pathways might be the reason for the above-mentioned studies observing an attenuated immune response as a result of endurance exercise.

As a conclusion, the presented studies show different results of miRNA expression depending on exercise duration but not type. Acute aerobic exercise in small doses induces anti-inflammatory response whereas exhaustive endurance exercise (such as a marathon) increases proinflammatory miRNAs [12,30,37]. It seems that the NF-κB pathway plays a crucial role in exercise-mediated inflammatory response. In de Gonzalo-Calvo’s study, miR-150-5p targeted the PI3K/Akt pathway and subsequently suppressed the NF-κB pathway after a 10 km run, whereas after a marathon, miR-146, which is known to enhance production of transcriptional factors of the NF-κB pathway, was upregulated [12,30]. Another important miRNA that takes part in exercise-dependent immune response is the let-7 family upregulating the toll-like receptor (TLR) pathway which initiates and develops inflammatory response. The let-7 family is upregulated in response to exhaustive endurance exercise, however, repetitive doses of aerobic exercises in training programs inhibit inflammation by downregulation of this family [30,32]. It seems that the NF-κB pathway, TLR pathway, and miRNAs related to them (miR-146, miR-150-5p, miR-21, miR-148, miR-223, let-7 family) are most important in exercise-dependent inflammatory response. However, more studies focusing on miRNAs and gene expression are needed to confirm such observations.

### 3.5. MicroRNAs and VO_2max_

Maximal oxygen uptake (VO_2max_) is defined as a maximal rate of oxygen consumption during incremental exercise. It is an indicator for integrated pulmonary, cardiovascular, and muscular capacity to uptake and utilize O_2_. Various exercises increase the VO_2max_ capacity, however, aerobic high-intensity training was proven to be the most effective [60]. VO_2max_ was identified as a strong predictor of cardiovascular death and all-cause mortality in healthy adults and in patients with CAD [61].

Elite endurance athletes present high VO_2max_ due to large left cardiac chamber capacity and subsequently high cardiac output. Several miRNAs were identified which were correlated with the parameters of aerobic performance. An exercise-induced increase of miR-1, miR-133a, miR-206, miR-208b, and miR-499 was demonstrated directly after a marathon run. Further analysis revealed that miR-1, miR-133a, and miR-206 were positively correlated with both VO_2max_ and running speed at individual anaerobic lactate threshold (VIAS) [11]. MicroRNA-1, miR-133a, and miR-206 are typical miRNAs enriched from muscles. MicroRNA-1 and miR-206 regulate differentiation of skeletal myoblasts and miR-1 and miR-133a are considered as antihypertrophic factors. Probably, this unique ability to influence both tissues makes the mentioned miRNAs suitable as biomarkers for cardiopulmonary fitness.

Denham et al. confirmed a potential role of miR-1 in aerobic performance capacity, and suggested that together with miR-486, they may constitute an independent predictor of VO_2max_. MicroRNA-1 and miR-486 were elevated and correlated significantly with the VO_2max_ values, and miR-486 was inversely correlated with heart rate at rest, after completing a four-week training program [18]. Moreover, inflammation-related miRNA such as miR-146a was correlated linearly with the increase of peak oxygen consumption before, as well as after, the training program. A similar expression pattern was observed with miR-20 indicating that both miR-146a and miR-20a could be plasma-based markers of cardiopulmonary fitness (see details in Table 1) [16]. Finally, 12 patients with the highest and 12 with the lowest VO_2max_ were selected out of 4631 patients from HUNT Fitness Study in order to perform the miRNA expression profiling. As a result, miR-210 was significantly higher in a validation cohort and therefore it was negatively correlated with VO_2max_ values. MicroRNA-21 and miR-222 presented weak correlation with VO_2max_, however, their value was considered predictive of CVD [33].

Kern et al. showed that miRNA expression can be altered due to regular endurance training and highlighted six possible miRNA clusters that can be utilized to predict phenotypic VO_2max_ levels, where clusters 1, 2, and 6 were mostly depleted in the top list of negative feature coefficients (see Table 1). Furthermore, it was suggested that miR-532-5p could be a potential biomarker of VO_2max_ changes after carbohydrate uptake. It is in line with their miRNA enrichment analysis findings as VO_2max_ was significantly associated with the insulin signaling pathway [35].

In conclusion, miR-1, miR-133, miR-206, miR-146a, miR-20 were positively correlated with the VO_2max_ values, which suggests their significance as potential biomarkers for cardiopulmonary fitness. Interestingly, discrepant results were observed regarding miR-486 and its correlation with VO_2max_. These inconsistencies are likely to be the product of many factors that vary across studies: (1) differences in participants (e.g., healthy endurance athletes who trained more than three times per week for at least one year versus young healthy subjects with no regular exercise regimen); (2) differences in training protocols (e.g., short-term VO_2max_ ergometer test versus long-term steady-state cycling program); (3) differences in blood samples collection schedules; and (4) differences in data analysis. In view of these many differences, it is not surprising that the literature is inconsistent. More studies should be conducted to estimate miR-486 value as a biomarker of cardiopulmonary fitness.

## 4. Conclusions

In the present article, we summarized the current knowledge of miRNA involvement in endurance training comprehensively, and also discussed the miRNAs that are regulated upon cardiac hypertrophy, cardiac myocyte damage, fibrosis, and inflammatory response during and after the endurance training. After a detailed literature search, we found that miR-1, miR-133, miR-21, and miR-155 are crucial in adaptive response to exercise. Studies available in the literature showed different effects on inflammatory-related miRNA expression depending on exercise duration but not its type [30]. Further studies are needed in order to determine interactions between miRNAs and genes involved in adaptive changes depending on the type and duration of the training.

## Abbreviations

ACS	acute coronary syndrome
CAD	coronary artery disease
c-miRNA	circulating microRNA
CRP	c-reactive proteins
CTGF	connective tissue growth factor
CVD	cardiovascular disease
GDF1	growth differentiation factor 1
HCM	hypertrophic cardiomyopathy
hs-cTnT	high-sensitivity cardiac troponin T
ISH	ischemic heart disease
LA	left atrium
LV	left ventricle
MACE	major adverse cardiac events
min/week	minutes per week
miR	microRNA
MMP	matrix metalloproteinase
mRNA	messenger RNA
NT-pro-BNP	n-terminal b-type natriuretic peptide
RNA	ribonucleotide acid
SCD	sudden cardiac death
TIMP	tissue inhibitor of metalloproteinase
TLR	toll-like receptors
WHO	World Health Organization
